# Study of Physical and Degradation Properties of 3D-Printed Biodegradable, Photocurable Copolymers, PGSA-*co*-PEGDA and PGSA-*co*-PCLDA

**DOI:** 10.3390/polym10111263

**Published:** 2018-11-13

**Authors:** June-Yo Chen, Joanne V. Hwang, Wai-Sam Ao-Ieong, Yung-Che Lin, Yi-Kong Hsieh, Yih-Lin Cheng, Jane Wang

**Affiliations:** 1 Department of Chemical Engineering, National Tsing Hua University, Hsinchu 30013, Taiwan; juneyochen@gapp.nthu.edu.tw (J.-Y.C.); joanne8401025@gmail.com (J.V.H.); namelessandsam@yahoo.com.tw (W.-S.A.-I.); j29462013@gmail.com (Y.-C.L.); d944530@oz.nthu.edu.tw (Y.-K.H.); 2 Department of Biomedical Engineering and Environment Sciences, National Tsing Hua University, Hsinchu 30013, Taiwan; 3 Department of Mechanical Engineering, National Taiwan University of Science and Technology, Taipei 10607, Taiwan; ylcheng@mail.ntust.edu.tw; 4 R&D Center for Membrane Technology, Chun Yuan Christian University, Taoyuan 32023, Taiwan

**Keywords:** biodegradable polymer, photocurable polymer, additive manufacturing, digital light processing, poly(glycerol sebacate) acrylate, polycaprolactone diacrylate, poly(ethylene glycol) diacrylate

## Abstract

As acrylated polymers become more widely used in additive manufacturing, their potential applications toward biomedicine also raise the demand for biodegradable, photocurable polymeric materials. Polycaprolactone diacrylate (PCLDA) and poly(ethylene glycol) diacrylate (PEGDA) are two popular choices of materials for stereolithography (SLA) and digital light processing additive manufacturing (DLP-AM), and have been applied to many biomedical related research. However, both materials are known to degrade at a relatively low rate in vivo, limiting their applications in biomedical engineering. In this work, biodegradable, photocurable copolymers are introduced by copolymerizing PCLDA and/or PEGDA with poly(glycerol sebacate) acrylate (PGSA) to form a network polymer. Two main factors are discussed: the effect of degree of acrylation in PGSA and the weight ratio between the prepolymers toward the mechanical and degradation properties. It is found that by blending prepolymers with various degree of acrylation and at various weight ratios, the viscosity of the prepolymers remains stable, and are even more 3D printable than pure substances. The formation of various copolymers yielded a database with selectable Young’s moduli between 0.67–10.54 MPa, and the overall degradation rate was significantly higher than pure substance. In addition, it is shown that copolymers fabricated by DLP-AM fabrication presents higher mechanical strength than those fabricated via direct UV exposure. With the tunable mechanical and degradation properties, the photocurable, biodegradable copolymers are expected to enable a wider application of additive manufacturing toward tissue engineering.

## 1. Introduction

Acrylated polymers are highly popular in the glue [1,2] and coating [3,4,5] industry. They are well known for the crosslinkability between the double bonds and their fast reaction rates. The radical polymerization that acrylated polymers go through generally require an initiation process, which may be triggered by heat, catalyst, or light [6,7]. The rapid reaction and photocurable properties of some of the acrylated polymers have also led to their applications toward 3D printing [8,9].

Stereolithography (SLA) and digital light processing additive manufacturing (DLP-AM) are two of the 3D printing methods involving radical polymerizations triggered via light. Both of the fabrication methods are considered highly precise and efficient compared to fused deposition modeling (FDM) [10,11]. Unlike FDM, which relies on the extrusion of molten thermoplastics, SLA and DLP-AM initiates the crosslinking between acrylated polymer chains via the exposure to light at various wavelengths. SLA is known for the use of single laser beam (UV) as the light source, while DLP-AM employs the digital mirror device (DMD) to control the exposure of light in an array simultaneously [12]. SLA is known to be more time-consuming, but capable of high resolutions as low as 2 μm [11]. Meanwhile, DLP-AM systems are capable of rapid prototyping and are cheaper than typical SLA systems due to the wider options in light sources [13]. Both additive manufacturing methods have gained lots of attention and opened up new markets over the past decades. However, the brittle nature and toxicity of the acrylated polymers limited the applications of the two systems toward the biomedical field, especially in tissue engineering. More and more attention has been focused on the development of biocompatible or biodegradable acrylates over the years [14,15,16].

In the search for acrylated polymers that may be applied in biomedicine, many studies have been dedicated to the conversion of existing biocompatible/biodegradable polymers—such as poly(lactic-*co*-glycolic acid) (PLGA), polycaprolactone (PCL), and polyethylene glycol (PEG)—into photocurable polymer by introducing acrylate groups to the polymers. In the work of Ge, et al., PLGA has been applied in the fabrication of 3D printed scaffolds and cultured with human fetal osteoblasts [17] and osteosarcoma cells [18] for bone tissue regeneration. PEG has also been converted into polyethylene glycol diacrylate (PEGDA), which are readily purchasable, for various applications, including scaffolds for cell culture with bone marrow stromal cells [19] and human mesenchymal stem cells [20], and scaffolds for aortic valve [21]. Polycaprolactone diacrylate (PCLDA) have also been synthesized and printed via LCD-projected maskless additive manufacturing system by Cheng, et al. [22]. The emergence of the biodegradable, photocurable polymers have brought forth new options toward the fabrication of biomedical devices and scaffolds. However, photocurable polymeric materials are often criticized for their high Young’s modulus and their reduced degradation rate, limiting their applications to bone tissue engineering, and less compatible for soft tissue engineering [23,24]. Meanwhile, gelatin methacryloyl (GelMA) have been introduced as a photocurable hydrogel for tissue engineering, and have been shown 3D printable via proposed projection stereolithography [25]. Yet the low Young’s modulus and fast degradation also limits the applications of GelMA to vascularization and cell culture [26].

Poly(glycerol sebacate) (PGS) was first developed in MIT in 2002, known as the biorubber [27]. Its high elasticity and low Young’s modulus along with the bioabsorbability made it a good choice of material for soft tissue engineering, such as heart [28] and arterial [29]. In 2007, photocurable, biodegradable polymer poly(glycerol sebacate) acrylate (PGSA) is introduced by adding acrylate groups to PGS by Nijst, et al. [30]. Compared to PGS, which is an elastomer that typically requires a secondary cure ranging between 8–48 h [27,31,32]. PGSA is a photocurable elastomer which may be cured via UV curing at room temperature for 10 min [30]. In this work, we propose the co-polymerization of PGSA and PEGDA or PCLDA at various degree of acrylation and ratio to provide a wide range of selection of biodegradable, photocurable polymers for soft tissue engineering. Through the combination of two or three of the abovementioned prepolymers, the mechanical and degradation properties can be easily modified. If combined with suitable blending, delivery, and curing setups, scaffolds with varying mechanical properties maybe fabricated through 3D printing.

## 2. Materials and Methods

All materials were purchased from Sigma-Aldrich (St. Louis, MO, USA), and used as received unless otherwise specified.

### 2.1. Synthesis of PGSA Prepolymer

PGSA prepolymer was synthesized according to previously published methods [27,30]. An equimolar mixture of glycerol and sebacic acid was added into a two-neck round-bottom flask and melted at 130 °C under nitrogen for 2 h. PGS prepolymer was formed via polycondensation at 130 °C under low pressure for 24 h and cooled to room temperature for further usage. PGS acrylation was performed as follows: 30 g of PGS prepolymer, 30 mg of 4-(dimethylamino)pyridine (DMAP) (Alfa Aesar, Ward Hill, MA, USA), and 300 mL of dichlormethane (DCM) (Echo Chemical Co., Ltd., Miaoli, Taiwan) were mixed in a two-neck round-bottom flask under nitrogen for 10 min before the reaction was cooled to 0 °C. Then triethylamine (125 mol% of acryloyl chloride amount) (Alfa Aesar, Ward Hill, MA, USA) was added, followed by slow addition of acryloyl chloride (60, 30, and 15 mol % of hydroxyl groups on PGS prepolymer for synthesis of PGSA30, 15 and 7 respectively). (Merck & Co., Kenilworth, NJ, USA), and reacted at room temperature for 24 h. The resulting mixture was rotary-evaporated, dissolved in ethyl acetate (EA) (Echo Chemical Co., Ltd., Miaoli, Taiwan) and filtered repeatedly. After mixing with 5 × 10^−6^ M hydrochloric acid in a separating funnel and settled for 24 h, the prepolymer solution was pipetted out and EA was removed to obtain the PGSA prepolymer. All PGSA prepolymers are characterized via VNMRS-700 NMR spectrometer (Varian, Palo Alto, CA, USA) for the degree of acrylation. The calculation and NMR spectra are shown in Figure S1 in the Supplemental Material. The calculated degree of acrylation in PGSA (DA = 30%) is abbreviated as PGSA30 throughout this work. Similarly, PGSA (DA = 15%) is PGSA 15 and PGSA (DA = 7%) is PGSA 7.

### 2.2. PCLDA Prepolymer Synthesis

Polycaprolactone diacrylate (PCLDA) was synthesized according to the work of Kweon, et al. [33] in the following steps. 4 g (0.002 mol) of polycaprolactone diol (*M*_n_ 2000) and 40 mL of toluene (Alfa Aesar, Ward Hill, MA, USA) were mixed in a two-neck round-bottom flask under nitrogen. After fully dissolved, nitrogen was stopped and 0.63 mL (0.0045 mol) of triethylamine and 0.37 mL (0.0045 mol) of acryloyl chloride were added, respectively. The reaction was performed at 80 °C for 3 h before cooled down to room temperature. Then triethylamine hydrochloride was removed via filtration and 400 mL of n-hexane was added. PCLDA prepolymer precipitation was collected and dried at 50 °C for 24 h to remove the solvent. The NMR spectrum is of PCLDA synthesized is shown in Figure S2 in the Supplemental Material.

### 2.3. Prepolymer Blending

PGSA, PCLDA, and PEGDA (*M*_n_ 700) prepolymers were mixed with different weight percent ratios and stirred for 30 min to obtain fully mixed materials. 3 wt % of 2,4,6-trimethyl benzoyl diphenyl phosphine oxide (TPO) was added to the mixtures as photoinitiator to accelerate the photopolymerization reaction.

### 2.4. Polymer Rheological Properties

The rheometer MCR 302 (Anton Paar, Graz, Austria) was used to measure the viscosity of prepolymer mixture. The analysis was performed in parallel plate geometry (*d* = 25 mm; gap = 0.2 mm) at shear rate ranging from 0.1 to 1000 s^−1^. The measuring temperature for PCLDA prepolymer was set at 40 °C [34], while others were set at 25 °C.

### 2.5. DLP-AM Printing of Polymer Film/Mechanical Tester

Digital light processing system was assembled by the Cheng lab at National Taiwan University of Science and Technology, and printing protocol was modified based on previous published methods [22,35]. Briefly, STL files created by SOLIDWORKS^®^ 2017 were processed by computer to create patterns for each layer. Prepolymer mixtures were loaded onto the loading platform where the projector (Acer, New Taipei, Taiwan) projected patterns to cure the material. After one layer was cured, the probe moves upward in *z*-axis for next-layer exposure. The structure was printed layer-by-layer and removed from the probe after printing. Printed parts were washed with 95% ethanol and dried at room temperature for further tests.

### 2.6. Mechanical Property

Mechanical testers (30 × 3 × 0.5 mm^3^) (*n* = 5), prepared by DLP-AM and UV-curing respectively, were tested on an TA-ElectroForce^®^ 3200 Series III system (Thermal Analysis, New Castle, DE, USA) and a 225 N load cell. Specimens were extended at a rate of 0.08 mm/min until break. Ultimate tensile strength and elongation at break data were collected, and Young’s modulus (MPa) was calculated from the slope of the first 10% of the stress–strain curve. A detailed analyses of the thermal properties of the prepolymers and copolymers are shown in Figure S3 and Table S1 in the Supplemental Material.

### 2.7. Degradation Property

Degradation samples (diameter = 1.5 cm; thickness = 1 mm) (*n* ≥ 3) were prepared by DLP-AM and soaked subsequently into ethanol solutions with the following concentrations for 12 h each: 95%, 75%, 50%, 25%, and 0% (RO water). Samples were then dried at 50 °C for seven days and weighed before tests. Enzyme degradation was carried out according to Hsieh, et al. [36] and Lin, et al. [37]. In short, lipase from porcine pancreas Type II was dissolved in phosphate buffer solution (20 units/mL) and filtrated before use. Degradation samples and 4 mL of enzyme solution were placed in 12-well cell culture plates. Then the enzyme degradation was carried out at 37 °C with enzyme solutions changed every two days. Samples were collected, washed with distilled water, dried at 50 °C for seven days and weighed again to determine the mass loss.

### 2.8. Swelling Ratio

After the degraded samples were washed, dried and weighed. The samples were soaked in PBS solution at 37 °C for 24 h and weighed again to determine the wet weight. The swelling ratio was calculated from the equation [38]

(1) $$\;\mathrm{Swelling}\;\mathrm{Ratio}=\;\frac{wet\;weight-dry\;weight}{dry\;weight}\times 100\%\;$$

### 2.9. NaOH Degradation

NaOH degradation was modified from the protocols of Lam, et al. [39] with modifications to the sample sizes and NaOH concentration. Degradation samples (diameter = 1 cm; thickness = 0.5 mm) (*n* ≥ 3) were prepared by DLP-AM, and soaked subsequently into ethanol solutions with the following concentrations for 12 h each: 95%, 75%, 50%, 25%, and 0% (RO water). Samples were then dried at 50 °C for seven days to determine the dry weight. Two groups of dried samples were soaked into 0.1 M NaOH solution at 37 °C for 24 and 48 h respectively. After degradation, samples were washed with RO water, dried at 50 °C for seven days and weighed again to determine the mass loss.

### 2.10. UV Film Formation

Prepolymer blends were pipetted into metal molds and covered with microscope slides (1 mm thick) on each side. Then the assembly was exposed to ultraviolet light (UV curing box, Hg lamp with wavelength 365 nm, ~12 mW/cm^2^) for 30 s per side. Polymer films (70 mm × 20 mm × 0.5 mm) were obtained and further prepared into proper dimension for degradation and mechanical characterizations.

## 3. Results and Discussion

### 3.1. Prepolymer Blends

#### 3.1.1. Study of Prepolymer Miscibility

**Figure 1:**
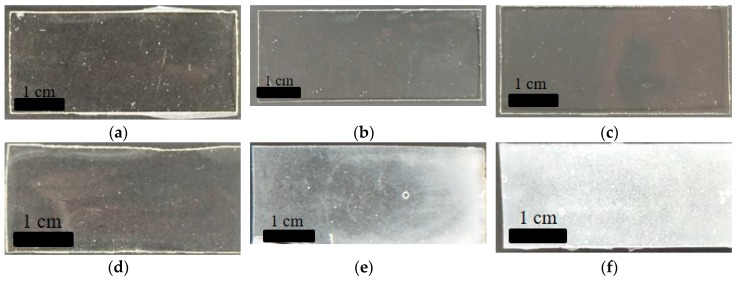
Visual observation of PGSA30-co-PEGDA in (**a**) 2:1, (**b**) 1:1, (**c**) 1:2 ratio, and PGSA30-*co*-PCLDA in (**d**) 2:1, (**e**) 1:1, (**f**) 1:2 ratio.

In order to prepare the resin for 3D printing via DLP-AM, polymer blends of PGSA, PEGDA, and PCLDA prepolymers were prepared. During the blending process of the prepolymers, it was found that PGSA and PEGDA prepolymer are 100% miscible, and upon crosslinking under UV light, forms transparent films (Figure 1a–c) regardless of the mixing ratio. Contrary to the high miscibility between PGSA and PEGDA, PCLDA is much less miscible with PGSA. White precipitations were observed in the polymer films with increasing ratio of PCLDA (Figure 1d–f). Two factors may contribute to this phenomenon: backbone hydrophilicity and molecular weight of prepolymer. First, although the sebacic acid in PGSA contains a longer hydrocarbon chain, the glycerol compensates for the missing hydrophilicity. Compared to the well-known hydrophilicity of PEGDA, PCLDA is of a much more hydrophobic nature. Second, the molecular weight of PGSA, PEGDA, and PCLDA are about 5000, 600, and 2000 g/mol, respectively. Since both PGSA and PCLDA are larger molecules, the blending process may have been hindered by the entanglement between the long chains of PGSA prior to polymerization. In contrast, the short PEGDA may behave like a solvent when mixed with PGSA. Through the miscibility tests, it was discovered that although PGSA mixes well with PEGDA at all weight ratio, it is only capable of dissolving PCLDA up to about 33 wt %. Therefore, the ratio between PGSA and PCLDA were set at 2:1 to avoid precipitation from insolubility of PCLDA, while PGSA and PEGDA were mixed at 1:1: ratio for the general characterization.

#### 3.1.2. Characterization of Rheological Properties of Copolymer Blends

**Figure 2:**
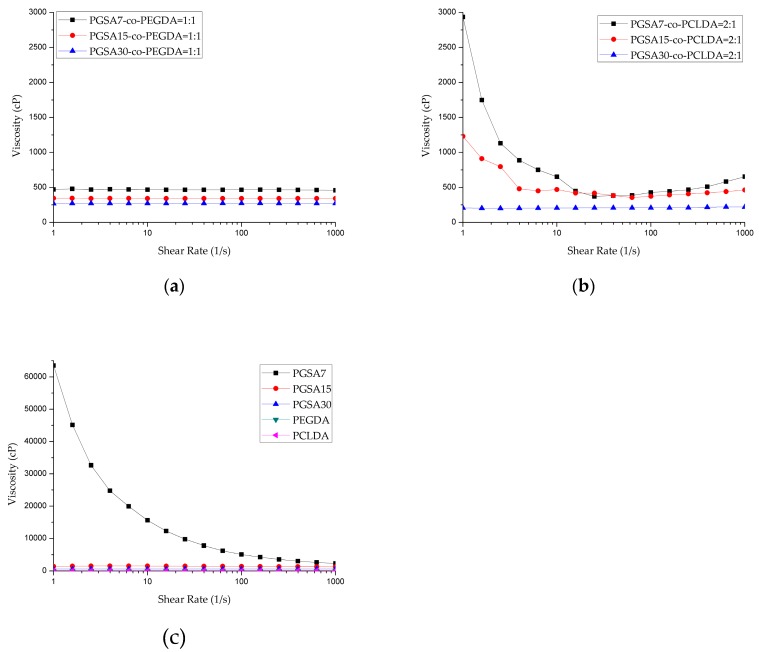
Apparent viscosity as a function of shear rate for (**a**) PGSA-*co*-PEGDA in various degree of acrylation; (**b**) PGSA-*co*-PCLDA in various degree of acrylation; (**c**) PEGDA, PCLDA, and PGSA prepolymers in various degree of acrylation. Note that PCLDA was measured at 40 °C instead of 25 °C according to the printing parameter of Cheng et al. [34].

In order to ensure the printability of the polymer blends on the DLP-AM system, it is critical to ensure that the viscosity is within printable range, which is typically between 250–1000 cP, with some exceptions that can go as high as 10,000 cP, and may be difficult to print [40,41]. Characterization of the rheological properties of the copolymer blends are shown in Figure 2, which indicates that most of the prepolymers in this work behave like Newtonian fluid, with the exception of PGSA7 and PGSA7-*co*-PCLDA prepolymers, which present sheer-thinning behaviors. The average viscosity is summarized in Table 1. It is noted that as the degree of acrylation decreases in PGSA prepolymers, the prepolymers become very viscous. The high viscosity in PGSA prepolymer makes it very difficult to print on DLP-AM, and the addition of solvents becomes necessary for the adjustment of viscosity. However, the addition of solvent may affect the precision and quality of printed scaffolds, and the remnant of solvents in the printed scaffolds may later create adverse effect in biomedical applications. Meanwhile, the low viscosity observed in PEGDA also makes it difficult to print precisely, with the fluidic prepolymer solution. In contrast, by combining PGSA with either PEGDA or PCLDA, regardless of the degree of acrylation in PGSA, the viscosity drops to workable ranges. By varying the degree of acrylation in PGSA while holding the weight ratio against PEGDA or PCLDA constant, it is clear that the viscosity of the prepolymer mixtures decreases slightly with an increasing degree of acrylation, while remaining in the workable range. The addition of PGSA at various degree of acrylation to linear, photocurable, biodegradable polymers may, therefore, bring about a range of novel photocurable, biodegradable copolymers that may be printed under similar 3D-printing conditions.

### 3.2. Formation of PGSA-co-PEGDA and PGSA-co-PCLDA Copolymeric Films with PGSA at Various Degree of Acrylation

#### 3.2.1. Proposed Mechanism for Photocurable Network Polymer Formation

**Figure 3:**
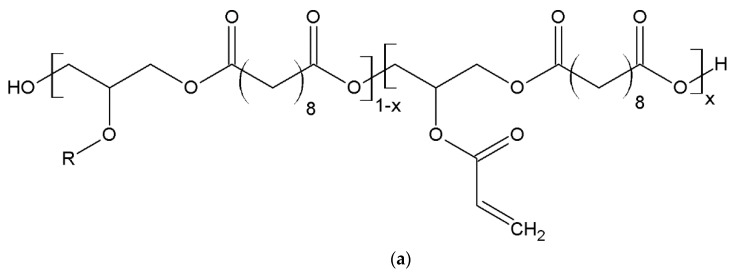

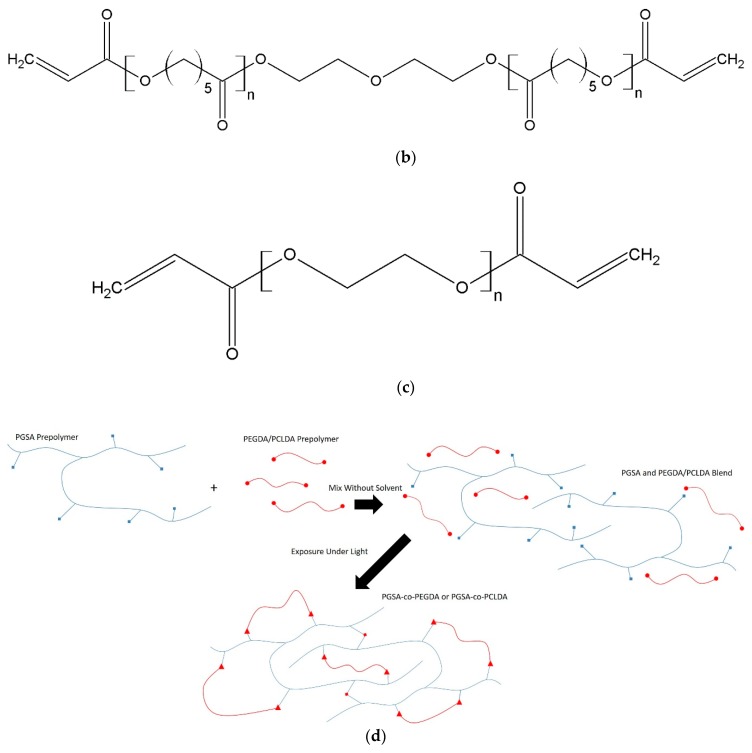
(**a**) General structure of PGSA where x indicates the degree of acrylation. (**b**) Structure of PCLDA and (**c**) PEGDA. (**d**) Sketch of the blending of PGSA prepolymer with PEGDA or PCLDA prepolymer, and the photocuring for the formation of PGSA-*co*-PEGDA or PGSA-*co*-PCLDA network polymers.

In the polymerization of photocurable polymeric materials, degree of acrylation is the key to the formation of polymer crosslinkages, and is known to directly influence the mechanical properties. For biodegradable, photocurable polymers, not only do the mechanical properties alter with crosslinking densities, the degradation properties are expected to alter as well. PEGDA and PCLDA are two of the most commonly used biodegradable, photocurable polymers in DLP-AM. As both PEGDA and PCLDA are linear polymers with acrylate groups attached to the two ends of the polymer, typical methods to change the crosslinking densities of the photocured polymers are through changing the molecular weights of the prepolymers. However, as the prepolymer molecular weight is altered, the polymer viscosity will increase accordingly, which may greatly limit their applications in 3D printing [41,42]. As an alternative, a series of copolymers are introduced in this work via the addition of biodegradable polymer PGSA to the formation of copolymers with PEGDA and/or PCLDA. Schematics of the PGSA, PCLDA, and PEGDA prepolymer structures are shown in Figure 3a–c. Since acrylate groups are attached to the hydroxyl groups on PGSA, the degree of acrylation is easily modified in PGSA prepolymers without altering the molecular weight of the prepolymers. PGSA prepolymers are blended with PEGDA and PCLDA and copolymerized via photocrosslinking, as shown in Figure 3d. For the photocrosslinking of pure PEGDA or PCLDA, since both polymers are linear, crosslinkages via radical polymerization would result in chain extension and physical entanglements. However, in the case of PGSA-*co*-PEGDA or PGSA-*co*-PCLDA, it is a combination between network polymer and linear polymer, creating chemically linked networks which are thermally stable and mechanically tunable. In this work, two main parameters are discussed regarding the tunable properties of the copolymers: degree of acrylation and mixing ratio, both of which are critical to varying crosslinking densities in the formation of co-polymers. To investigate the effect of degree of acrylation in PGSA toward the copolymerized films, PGSA with degree of acrylation of 7%, 15%, and 30% were blended with PEGDA and PCLDA and copolymerized via DLP-AM fabrication throughout this work.

#### 3.2.2. Study of Copolymer Mechanical Properties

Tensile tests were performed on the DLP-AM printed films and shown in Table 2. It is clear that the Young’s modulus and ultimate tensile strength (UTS) of PGSA-*co*-PEGDA and PGSA-*co*-PCLDA both increased with increasing degree of acrylation in PGSA at the same mixing ratio. The increases in Young’s moduli and UTS strongly indicate increases in crosslinking density of the polymer films, proving the mechanisms of the formation of network polymer proposed in Figure 3b. As the elongation at break is commonly in an inverse correlation to the Young’s modulus, it is clear that, with increasing degree of acrylation, the elongation at break decreases.

#### 3.2.3. Study of Copolymer Degradation Properties

**Figure 4:**
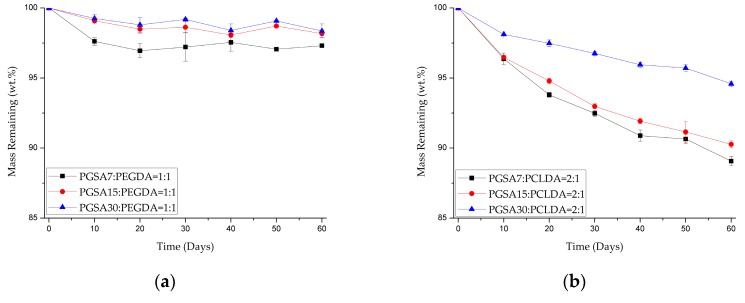
Mass remaining of (**a**) PGSA-*co*-PEGDA in 1:1 ratio and (**b**) PGSA-*co*-PCLDA in 2:1 ratio with various degree of acrylation degraded by 20 U/mL of lipase solution over 60 days at 37 °C.

In the degradation of photocurable, biodegradable polymers, two factors play dominating roles in changes in the degradation rate: degree of acrylation and the molecular weight of the polymer backbones. As the crosslinking densities increases for biodegradable polymers, it is anticipated that the degradation rate of the polymers will also decrease. Similarly, the degradation rate is also expected to increase with increasing molecular weight of prepolymers. In the work of Nijst et al. [30], full in vivo degradation of PGS was observed at six weeks, and PCL was observed to have degraded merely 7% in six months in the work of Lam, et al. [43]. With the addition of acrylation to the fabrication of scaffolds, the degradation of PGSA and PCLDA are expected to become even slower [30]. By combining PGSA with PEGDA and PCLDA, it is expected that the degradation rate will fall somewhere between the above rates. As shown in Figure 4, it is shown that, as the degree of acrylation further decreased from 30% to 15% and 7.5%, the degradation rate increased. Upon comparison of the degradation rate of the copolymers to that of the pure PEG and PCL polymers, the degradation rate of PGSA-*co*-PEGDA and PGSA-*co*-PCLDA is much faster.

**Figure 5:**
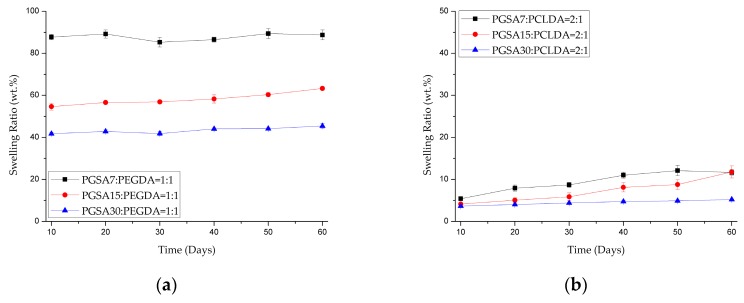
Swelling ratio of (**a**) PGSA-*co*-PEGDA and (**b**) PGSA-*co*-PCLDA over 60 days of degradation in 20 U/mL lipase solution at 37 °C. (*n* ≥ 3).

It is worth mentioning that the copolymerization of PEGDA with PGSA with varying degrees of acrylation also changes the swelling ratio of the copolymers dramatically, as shown in Figure 5a. For PGSA7-*co*-PEGDA, the swelling ratio was as high as 87.77% ± 1.32% after 10 days of degradation, while the swelling ratio quickly drops to 55% and 43% for PGSA15-*co*-PEGDA and PGSA30-*co*-PEGDA within the same 10 days. Compared to PGSA-*co*-PCLDA in Figure 5b, although the swelling ratio does decrease with increasing crosslinking densities, the effect is less prominent. This dramatic difference in the swelling ratio between PGSA-*co*-PEGDA and PGSA-*co*-PCLDA is likely the direct result of the strong difference in the hydrophilicity of the polymer backbones between PEGDA and PCLDA. For both of the copolymers, the changes in swelling ratio were all within 5% over 60 days of enzyme degradation, indicating that the degradation of the copolymers is dominated by surface erosion.

Since all PGSA30-containing films degraded at the lowest rate for all copolymers, a series of facilitated degradation was conducted by degrading PGSA30-copolymer films in 0.1 M of NaOH solution to further characterize the long term degradation of the copolymer films. The degradation results are shown in Table 3. As expected, since the PGSA backbone contains the highest amount of polyester linkages, pure PGSA polymers were completely degraded within the first 24 h, and PGSA30-*co*-PCLDA were completely degraded within 48 h. The degradation rate of PGSA30-*co*-PEGDA was similar to that of pure PEGDA films with only mild facilitation, while the PCLDA films barely degraded. This result also indicates that both the hydrophilicity and the degradability of the polymer backbones play critical rules to the long term degradation of the copolymers.

### 3.3. Effect of Prepolymer Mixing Ratio toward Copolymer Mechanical and Degradation Properties

#### 3.3.1. Study of Copolymer Mechanical Properties

As mentioned above, besides from varying the degree of acrylation in PGSA, another option to modify the crosslinking densities without altering the printing condition is to alter the mixing ratio of the prepolymers. As concluded earlier, although the miscibility between PGSA and PCLDA prepolymer is limited, PGSA and PEGDA prepolymers are highly miscible. Here, PGSA15 is chosen for the copolymers, and the mixing ratio between PGSA and PCLDA prepolymers are prepared at 2:1, 4:1, and 8:1, while PGSA and PEGDA are mixed at 2:1, 1:1, and 1:2. Tensile tests were performed on the DLP-AM printed films and shown in Table 4. As anticipated, as the content of PGSA decreases, both the Young’s modulus and UTS increases for the copolymers. It is also shown that with high PGSA contents, the elongation at break for PGSA15-*co*-PEGDA (2:1) and PGSA15-*co*-PCLDA (8:1) both fall around 18%, but as the PGSA content decreased, the elongation at break decreases. However, it is worth noting that the elongation at break of PGSA15-*co*-PEGDA (1:2) rebounded back to 26%. Considering the high elasticity of PGSA15 and PEGDA shown in Table 2, it is clear that with the higher presence of one particular prepolymer, it plays a more dominating role. The fact that copolymer blends at 1:1 ratio show a lower elongation at break than the other two copolymers indicates that the polymer chains may have somehow become less oriented after blending.

In this work, the average molecular weight of the PGSA synthesized is at about 5000 g/mol, which is composed of 17 repeating units, since each repeating unit of glycerol and sebacic acid is about 294 g/mol. Considering the 15% mole ratio of acrylation, an acrylate group is found for every 2000 Da in the PGSA prepolymers. Meanwhile, since the molecular weight of PEGDA and PCLDA are 700 and 2000 g/mol, each with two acrylate groups to the ends, an acrylate group is found for every 350 and 1000 Da in PEGDA and PCLDA prepolymers, respectively. Upon comparison of the prepolymers, the acrylate density in PEGDA is almost 2.14 time higher than PGSA, and the acrylate density in PCLDA is two times higher than PGSA. Therefore, it is possible to conclude that the increasing Young’s modulus of PGSA15-*co*-PEGDA and PGSA15-*co*-PCLDA over higher PEGDA and PCLDA content is a direct result of higher crosslinking densities in the prepolymers. Similarly, the large differences between the number of prepolymers available in the PGSA15-*co*-PEGDA (1:1) may have also played a role in the misalignment of the polymer chains and resulted in the low elongation at break.

#### 3.3.2. Study of Copolymer Degradation Properties

**Figure 6:**
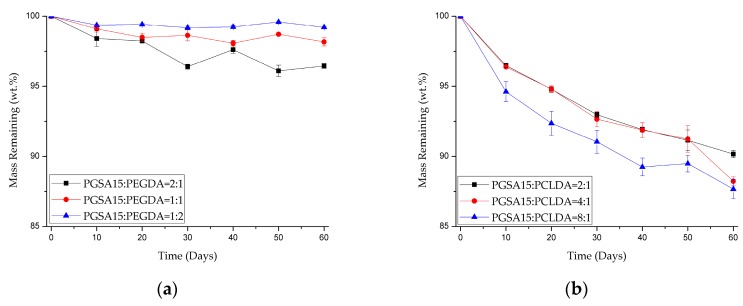
Mass remaining of (**a**) PGSA15-*co*-PEGDA and (**b**) PGSA15-*co*-PCLDA in various ratio degraded by 20 U/mL of lipase solution over 60 days at 37 °C.

Enzyme degradation of the copolymers were conducted over 60 days, and shown in Figure 6. It is observed that the degradation rate directly correlates to the ratio of PGSA in the copolymers. The higher the PGSA content, the faster the degradation rate. This is expected results, since PGSA degraded significantly faster than both PEGDA and PCLDA in NaOH solutions in Table 3. Overall, the results in Figure 4 and Figure 6, and Table 3 indicate that by adding PGSA to PEGDA and PCLDA, the degradation rate may be significantly increased, making the copolymers more suitable for the applications in soft tissue engineering than pure PEGDA and PCLDA.

### 3.4. Effect of Blending All Three Prepolymers toward Copolymer Mechanical Properties

As previously described in Figure 1, the miscibility of PCLDA in PGSA is rather limited. However, since PEGDA mixes with PGSA extremely well, it is then proposed that PEGDA be used as a solvent toward increasing the solubility of PCLDA in PGSA. In Table 5, the random copolymer of PGSA30-*co*-PEGDA-*co*-PCLDA in various ratio are prepared. With the addition of 20 wt % of PEGDA, up to 60 wt % of PCLDA were blended with 20 wt % of PGSA without precipitation. It is also noted that by holding PGSA content at 20 wt % (Sample A–C), the Young’s modulus is dominated by the content of PEGDA. Similarly, by holding PCLDA content at 20 wt % (Sample C, E, F), the Young’s modulus is also dominated by the content of PEGDA, making PEGDA content the dominant factor in Young’s modulus prediction. Upon comparison between PGSA and PCLDA by holding PEGDA content at 20 wt % (Samples A, D, F), it is found that although there is no particular dominance between the two in Young’s modulus, PCLDA plays a slightly more dominant role in UTS and elongation at break.

### 3.5. Comparison of Mechanical Properties between UV-Cured and DLP-AM Printed Copolymers

**Figure 7:**
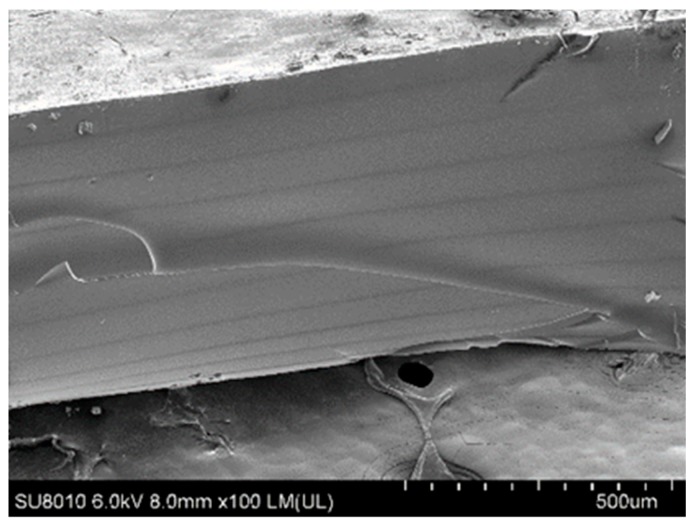
SEM imaging of the cross-section of a 10 layer DLP-AM printed PGSA30 scaffold.

As 3D printing, especially SLA and DLP-AM, become more widely accepted around the world, it is commonly praised for its high prevision and fast prototyping ability, but criticized for the brittle products. However, a comparison between UV-cured and DLP-AM printed copolymers were conducted and summarized in Table 6. Surprisingly, it is found that the DLP-AM printed copolymers all presented slightly higher mechanical properties than those cured under UV light. Meanwhile, the UTS of the copolymers from the two fabrication are also similarly higher in DLP-AM printed copolymers with one minor exception. In the fabrication of tensile test samples through DLP-AM, scaffolds are printed at 100 μm per layer for five layers. For each layer, the small distance between the light source and the reaction plate (100 μm) enables the penetration of light to trigger radical polymerizations, which may facilitate the exhaustion of the acrylate groups, thus resulting in higher crosslinking density per layer. Meanwhile, the UV-cured samples were radiated with UV light on both sides with possibly decaying UV-light exposure over the 500 μm thick prepolymer and the 1 mm thick glass slides on each side (totaling 2500 μm), hence leaving more unreacted acrylate residues. According to the mechanism proposed and proved through this work, higher crosslinking density is directly correlated to the Young’s modulus and UTS. Compared to the increasing Young’s moduli and UTS, the elongation at break is found much lower for DLP-AM fabricated copolymers. This is possibly due to the layer-by-layer exposure nature of DLP-AM fabrication. Through SEM imaging, it is shown in Figure 7 that dark lines are observed on the cross-sections of DLP-AM printed scaffolds each roughly 100 μm apart. Although the radical polymerization across the 100 μm thickness is well distributed, the interface between the printed layer and the newly formed layer maybe poorly attached, making the copolymers weaker at the interfaces.

## 4. Conclusions

In many existing 3D printers, multiple syringes or material inlets are often required for the fabrication of devices of multiple parts each with varying mechanical properties. However, the addition of new materials is often very costly, and the printing parameters often require modifications between different materials. By combining PGSA with PEGDA and/or PCLDA, it is now possible to print biodegradable polymers with similar composition, but very different mechanical and degradation properties, in a continuous motion through instantaneous blending of the three polymers at various ratios. This work aims to provide a data that describes the tunable mechanical and degradation properties for the selection of biodegradable, photocurable polymer that may become useful in 3D printing. The development of instantaneous blending of prepolymers while printing is already under way, and is hoped to bring evolutionary change to many existing 3D printing systems.

## Figures and Tables

**Table 1:** Average viscosity of PGSA, PEGDA, PGSA-co-PEGDA, and PGSA-*co*-PCLDA prepolymers with various degree of acrylation in comparison to PEGDA and PCLDA prepolymer (*n* = 3).

Polymer	Ratio	Viscosity (cP)
PGSA7	100%	Shear Thinning
PGSA15	100%	1401.14 ± 15.88
PGSA30	100%	594.97 ± 4.65
PGSA7-*co*-PEGDA	1:1	472.90 ± 43.02
PGSA15-*co*-PEGDA	1:1	343.87 ± 2.17
PGSA30-*co*-PEGDA	1:1	281.86 ± 5.50
PEGDA	100%	105.96 ± 9.32
PGSA7-*co*-PCLDA	2:1	Shear Thinning
PGSA15-*co*-PCLDA	2:1	422.82 ± 16.42
PGSA30-*co*-PCLDA	2:1	204.66 ± 5.86
PCLDA	100%	83.34 ± 6.63 *

* Measured at 40 °C instead of 25 °C according to the printing parameter of Cheng, et al. [34].

**Table 2:** Mechanical properties of DLP-AM printed PGSA, PEGDA, PGSA-*co*-PEGDA, and PGSA-*co*-PCLDA with various degrees of acrylation in comparison to PEGDA and PCLDA (*n* = 5).

Polymer	Ratio	Young’s Modulus (MPa)	Ultimate Tensile Strength (MPa)	Elongation at Break (%)
PGSA7	100%	0.12 ± 0.01 *	0.10 ± 0.01 *	121.23 ± 2.51 *
PGSA15	100%	1.55 ± 0.04 *	0.63 ± 0.01 *	46.95 ± 0.75 *
PGSA30	100%	5.10 ± 0.44	1.36 ± 0.08	28.43 ± 0.84
PGSA7-*co*-PEGDA	1:1	4.25 ± 0.40	0.80 ± 0.12	21.29 ± 1.73
PGSA15-*co*-PEGDA	1:1	7.58 ± 0.65	0.91 ± 0.07	13.63 ± 1.24
PGSA30-*co*-PEGDA	1:1	10.54 ± 0.82	1.10 ± 0.19	12.96 ± 2.25
PEGDA	100%	18.98 ± 1.11	3.19 ± 0.24	21.50 ± 2.19
PGSA7-*co*-PCLDA	2:1	1.42 ± 0.07	0.19 ± 0.01	22.39 ± 0.91
PGSA15-*co*-PCLDA	2:1	2.85 ± 0.30	0.20 ± 0.05	11.28 ± 3.22
PGSA30-*co*-PCLDA	2:1	7.00 ± 0.61	0.69 ± 0.08	14.08 ± 1.32
PCLDA	100%	4.35 ± 0.30	0.58 ± 0.10	15.34 ± 2.07

* Samples cured under UV radiation instead of DLP-AM.

**Table 3:** DLP-AM printed PGSA30, PGSA30-*co*-PEGDA, PEGDA, PGSA30-*co*-PCLDA, and PCLDA degraded in 0.1 M of NaOH at 37 °C.

Polymer	Ratio	Mass Remaining at 24 h (wt %)	Mass Remaining at 48 h (wt %)
PGSA30	100%	0.00 ± 0.00	0.00 ± 0.00
PGSA30-*co*-PEGDA	1:1	50.02 ± 0.57	39.38 ± 5.59
PEGDA	100%	52.22 ± 5.13	46.44 ± 6.16
PGSA30-*co*-PCLDA	2:1	41.95 ± 2.16	0.00 ± 0.00
PCLDA	100%	99.84 ± 0.23	99.85 ± 0.25

**Table 4:** Mechanical properties of DLP-AM printed PGSA15-*co*-PEGDA and PGSA15-*co*-PCLDA in various weight ratio (*n* = 5).

Polymer	Ratio	Young’s Modulus (MPa)	Ultimate Tensile Strength (MPa)	Elongation at Break (%)
PGSA15-*co*-PEGDA	2:1	4.66 ± 0.22	0.67 ± 0.05	18.41 ± 0.99
PGSA15-*co*-PEGDA	1:1	7.58 ± 0.65	0.91 ± 0.07	13.63 ± 1.24
PGSA15-*co*-PEGDA	1:2	9.03 ± 0.10	1.97 ± 0.07	25.94 ± 1.37
PGSA15-*co*-PCLDA	8:1	1.55 ± 0.10	0.14 ± 0.01	18.63 ± 1.63
PGSA15-*co*-PCLDA	4:1	2.30 ± 0.22	0.31 ± 0.03	19.72 ± 1.91
PGSA15-*co*-PCLDA	2:1	2.85 ± 0.30	0.20 ± 0.05	11.28 ± 3.22

**Table 5:** Mechanical properties of UV-cured PGSA15-*co*-PEGDA-*co*-PCLDA in various weight ratio (*n* ≥ 3).

Sample	Polymer	Ratio	Young’s Modulus (MPa)	Ultimate Tensile Strength (MPa)	Elongation at Break (%)
A	PGSA30-*co*-PEGDA-*co*-PCLDA	1:1:3	4.50 ± 0.27	1.37 ± 0.09	40.31 ± 2.70
B	PGSA30-*co*-PEGDA-*co*-PCLDA	1:2:2	5.56 ± 0.34	1.17 ± 0.06	27.81 ± 1.29
C	PGSA30-*co*-PEGDA-*co*-PCLDA	1:3:1	8.78 ± 0.68	1.22 ± 0.04	20.61 ± 0.78
D	PGSA30-*co*-PEGDA-*co*-PCLDA	2:1:2	4.84 ± 0.26	1.15 ± 0.09	36.74 ± 2.75
E	PGSA30-*co*-PEGDA-*co*-PCLDA	2:2:1	5.79 ± 0.42	1.11 ± 0.04	25.02 ± 1.18
F	PGSA30-*co*-PEGDA-*co*-PCLDA	3:1:1	3.84 ± 0.25	1.01 ± 0.03	35.10 ± 2.04

**Table 6:** Comparison between the mechanical properties of UV-cured and DLP-AM printed PGSA15-*co*-PEGDA and PGSA15-*co*-PCLDA in various weight ratio (*n* = 5).

Name	Ratio	Young’s Modulus (MPa)		Ultimate Tensile Strength (MPa)		Elongation at Break (%)	
		UV	DLP	UV	DLP	UV	DLP
PGSA15-*co*-PEGDA	2:1	4.22 ± 0.32	4.66 ± 0.22	0.61 ± 0.05	0.67 ± 0.05	18.42 ± 1.85	18.41 ± 0.99
PGSA15-*co*-PEGDA	1:1	7.54 ± 0.58	7.58 ± 0.65	0.90 ± 0.01	0.91 ± 0.07	14.58 ± 1.76	13.63 ± 1.24
PGSA15-*co*-PEGDA	1:2	8.59 ± 0.41	9.03 ± 0.48	1.12 ± 0.10	1.97 ± 0.07	19.87 ± 1.68	25.94 ± 1.37
PGSA15-*co*-PCLDA	2:1	1.88 ± 0.16	2.85 ± 0.30	0.57 ± 0.08	0.20 ± 0.05	44.40 ± 6.66	11.28 ± 3.22
PGSA15-*co*-PCLDA	4:1	0.95 ± 0.05	2.30 ± 0.22	0.31 ± 0.01	0.31 ± 0.03	45.95 ± 0.68	19.72 ± 1.91
PGSA15-*co*-PCLDA	8:1	0.67 ± 0.19	1.55 ± 0.10	0.18 ± 0.04	0.14 ± 0.01	41.46 ± 9.58	18.63 ± 1.63

## Supplementary Materials

The following are available online at http://www.mdpi.com/2073-4360/10/11/1263/s1, Figure S1: The ^1^H NMR spectra of (a) PGSA30, (b) PGSA15 and (c) PGSA7, Figure S2: The ^1^H NMR spectrum of PCLDA, Figure S3: The DSC analyses of (a) PGSA15, (b) PGSA30, (c) PGSA7-*co*-PEGDA = 1:1, (d) PGSA15-*co*-PEGDA = 1:1, (e) PGSA30-*co*-PEGDA = 1:1, (f) PEGDA, (g) PGSA7-*co*-PCLDA = 2:1, (h) PGSA15-*co*-PCLDA = 2:1, (i) PGSA30-*co*-PCLDA = 2:1 and (j) PCLDA. Table S1: Thermal properties of DLP-AM printed PGSA, PCLDA, PEGDA, and copolymer films.
