# Supplementary material for: Study of Physical and Degradation Properties of 3D-Printed Biodegradable, Photocurable Copolymers, PGSA-co-PEGDA and PGSA-co-PCLDA
Source: Polymers (Basel). 2018 Nov 13;10(11):1263. doi: 10.3390/polym10111263 (PMC6401713; doi:10.3390/polym10111263)
Supplement: Supplementary file 1 [file polymers-10-01263-s001.pdf]

polymers

Supplemental Material

^{1}H nuclear magnetic resonance (NMR) spectra of PGSA prepolymer were recorded using VNMRS-700 NMR spectrometer. The replacement of hydroxyl group by acrylate group is confirmed by the appearance of the peaks at 5.9, 6.2 and 6.4 ppm. The ester bond formation of secondary alcohol on tri-substituted glycerol is confirmed at 5.3 ppm and the resonance of protons from mono-substituted glycerol is confirmed at 3.7 ppm. The region of resonance of protons from sebacic acid is labeled on figure between 1—2.4 ppm. According to the work of Nijst et al., the degree of acrylation was calculated from the ^{1}H NMR[1]. In this work, the degree of acrylation was calculated by the following equation:

$$\text{Degree of acrylation} = \frac{\text{the average integral of acrylate gruop}}{\text{the average integral of sebacic acid}}$$

The degree of acrylation in PGSA30 is calculated by integral of acrylate group (1.000+0.774+1.071)/3 divided by the integral of sebacic acid (12.648)/4. Then the degree of acrylation in PGSA30 is 29.99%, as shown in **Figure S1 (a)**. In addition, the degree of acrylation in PGSA15 and PGSA7 are 14.38% and 6.85% respectively, and are shown as **Figure S1 (b)** and **Figure S1 (c)**.

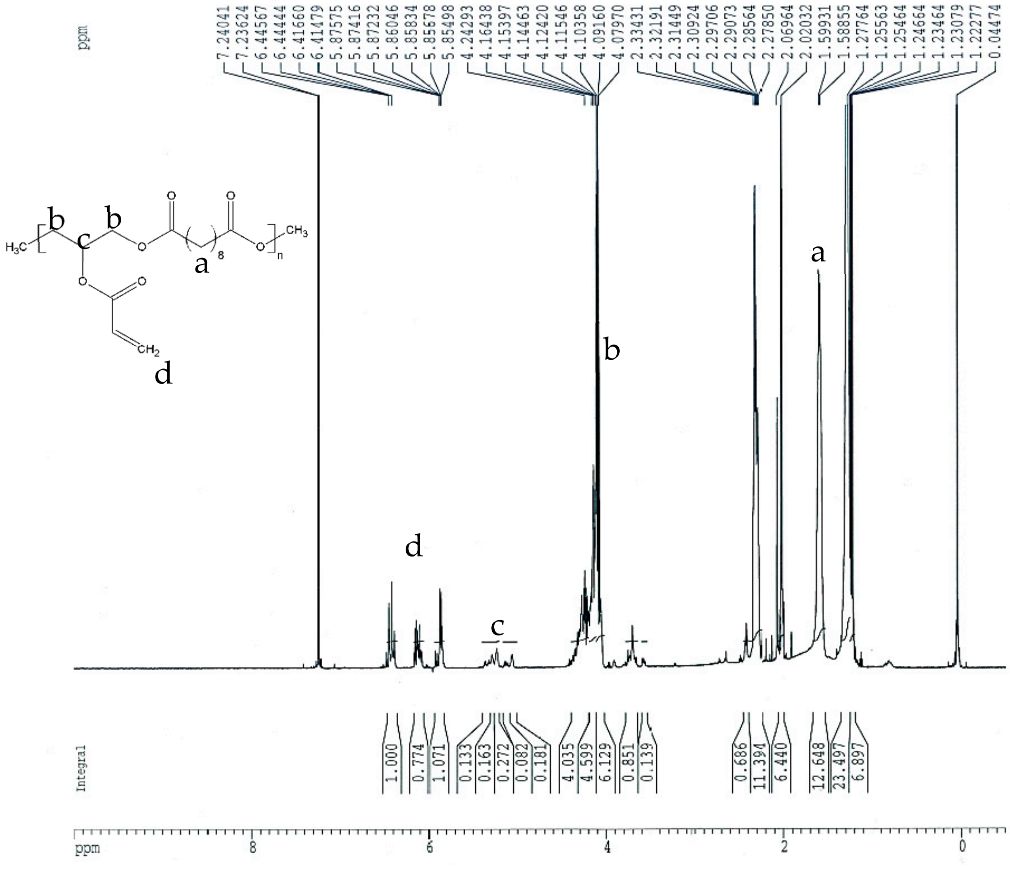

(a)

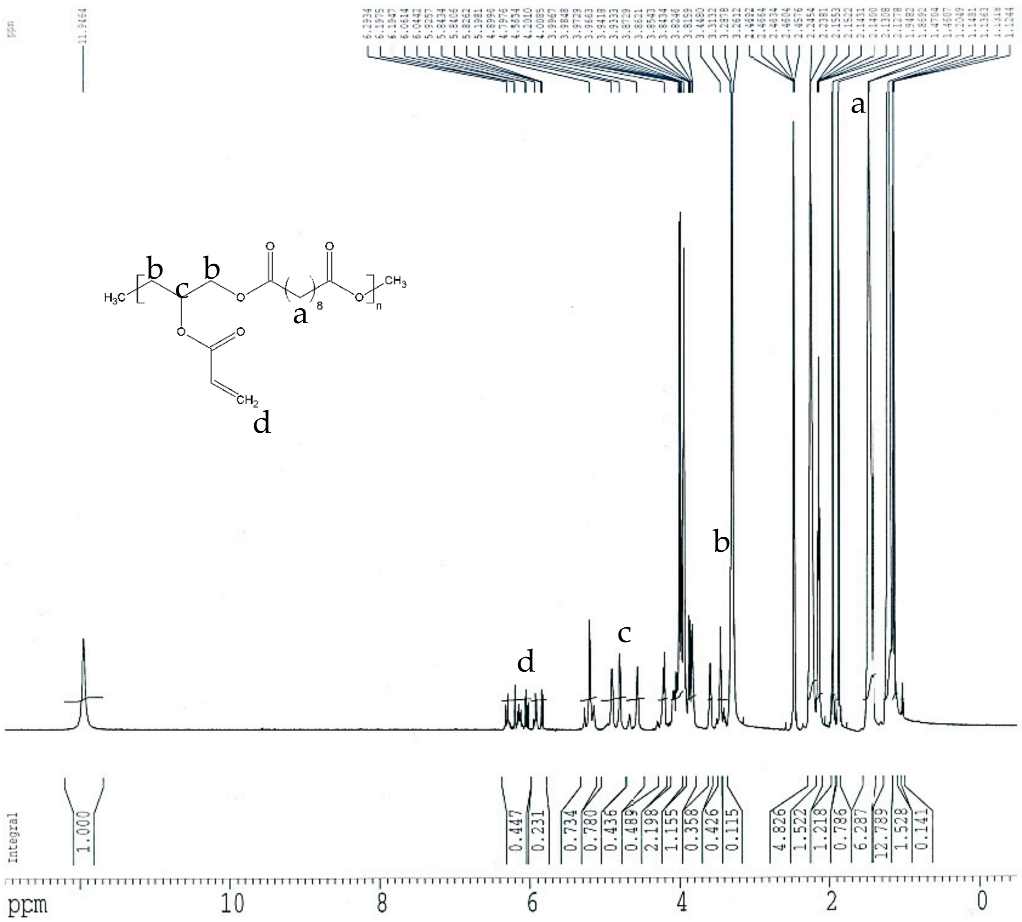
b
c
b
a
d
a
b
c
d
Integral
1.000
0.447
0.231
0.734
0.780
0.436
0.489
2.198
1.155
0.358
0.426
0.115
4.826
1.522
1.218
0.786
6.287
12.789
1.528
0.141
ppm
10
8
6
4
2
0

(b)

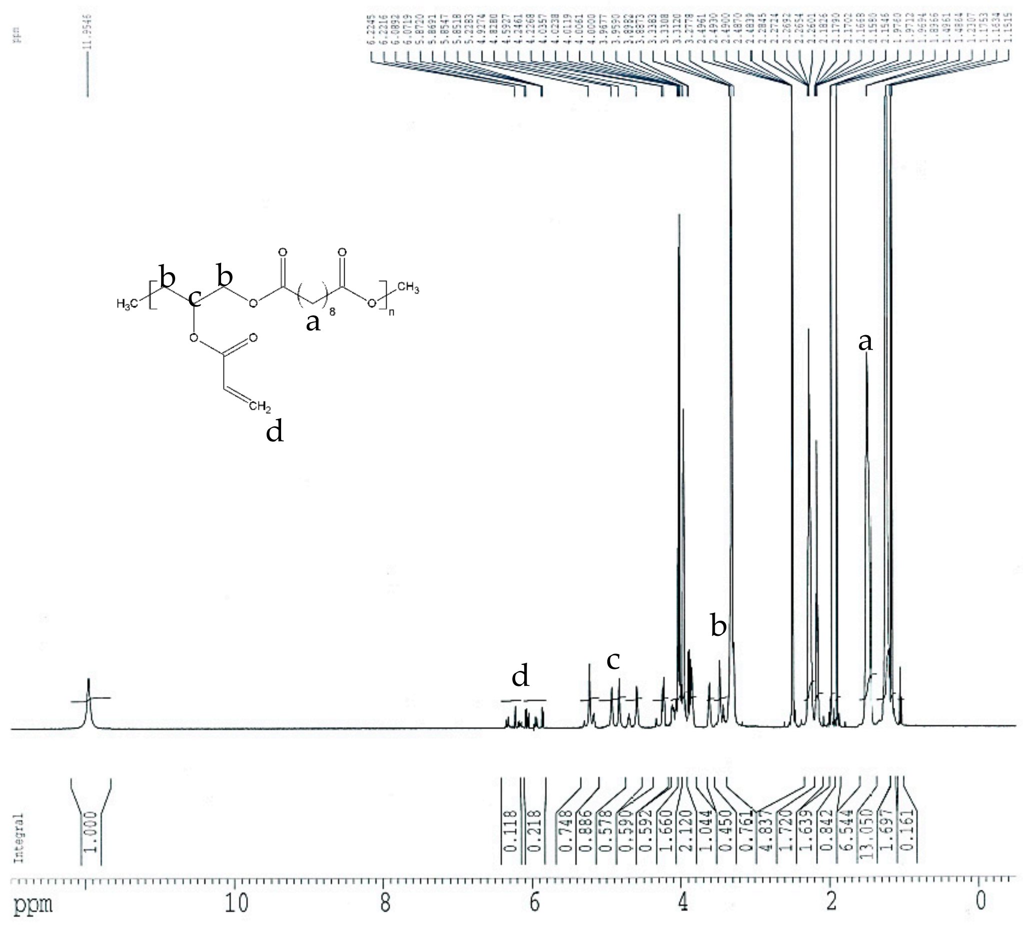

(c)

Figure S1 The ^{1}H NMR spectra of (a) PGSA30, (b) PGSA15 and (c) PGSA7, where sebacic acid is marked at region "a" and the hydrogen atoms on glycerol are shown in region "b" and "c". Region "d" represents the hydrogen atoms of the acrylate group on PGSA backbone, which indicates a successful synthesis.

In **Figure S2**, ^{1}H NMR analysis is also performed on the PCLDA synthesized.

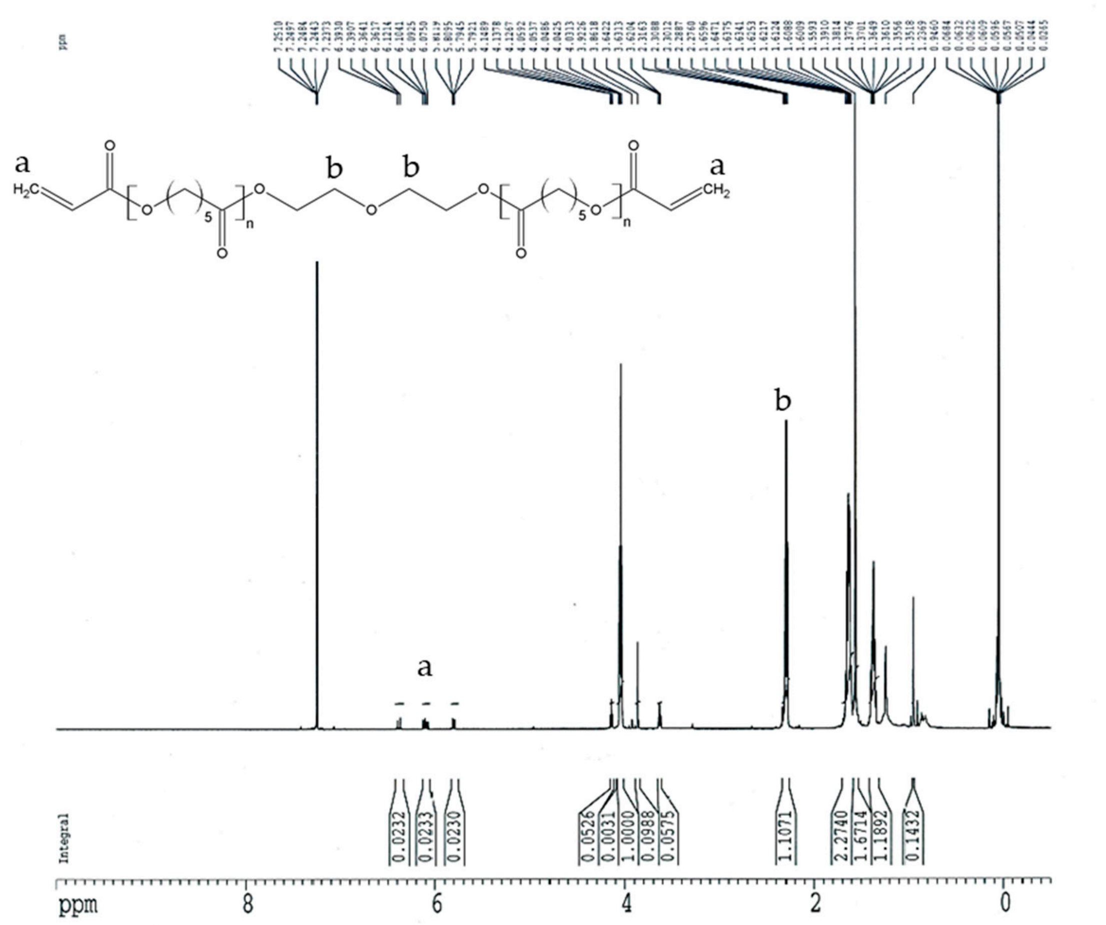

Figure S2 The ^{1}H NMR spectrum of PCLDA. The region "a" represents the hydrogen atoms of the acrylate group on PCLDA. On the other hand, region "b" indicates the hydrogen atoms on the PCLDA backbone.

The thermal properties of the DLP-AM printed films, including PGSA15, PGSA30, PCLDA, PEGDA and the polymer blends, were characterized through a TA-Q20 DSC (Thermal Analysis Co., USA). The measurements were carried out at a heating rate of 10 °C min^{-1} from -80 °C to 100 °C under a nitrogen flow. The DSC graphs are shown in **Figure S3** and summarized in **Table S1.**

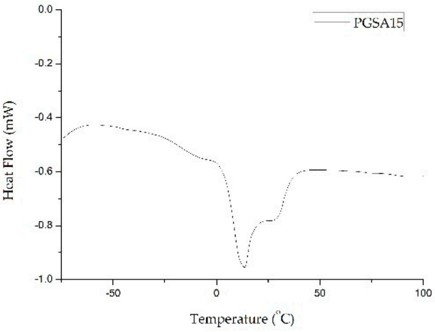

(a)

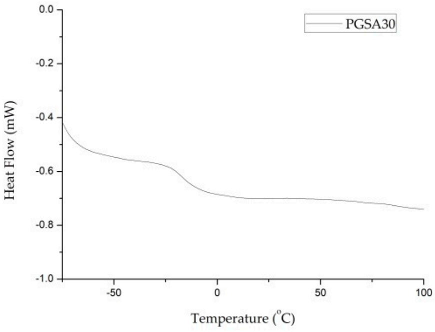

(b)

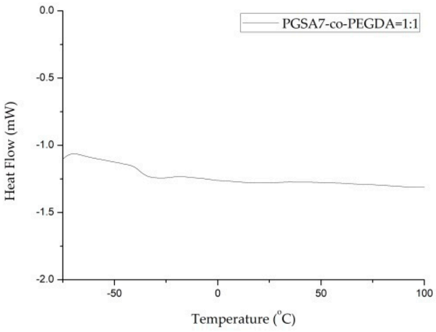

(c)

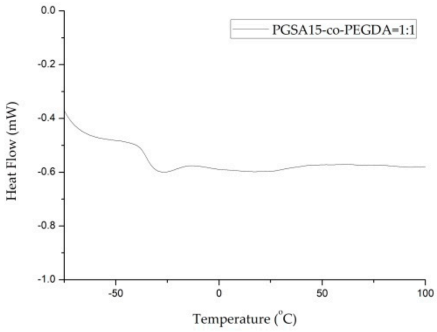

(d)

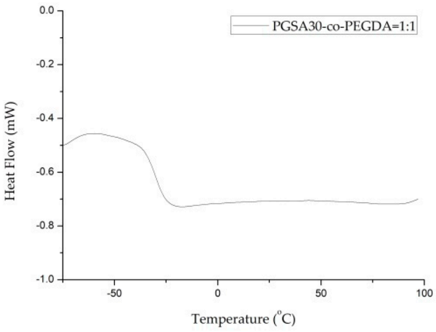

(e)

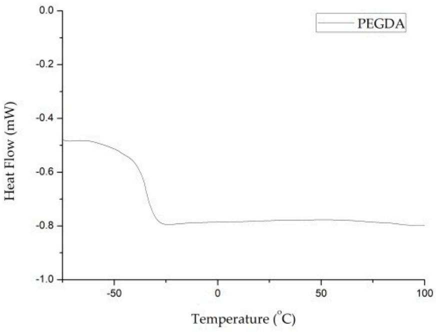

(f)

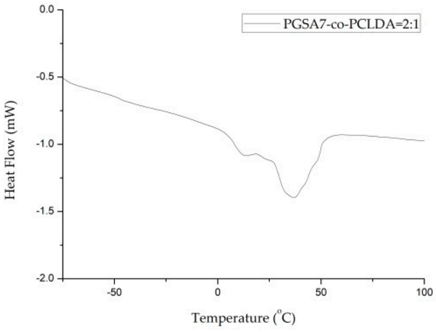

(g)

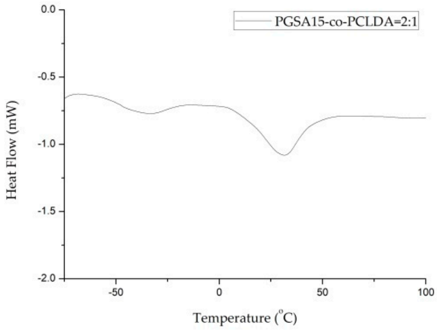

(h)

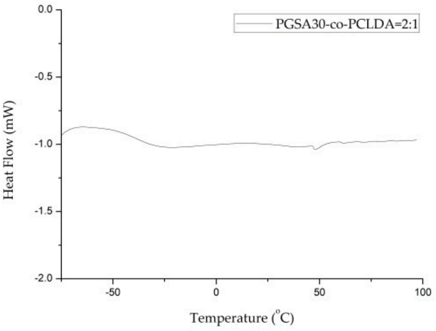

(i)

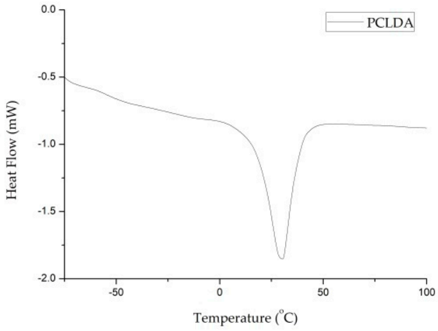

(j)

Figure S3. The DSC analyses of (a) PGSA15, (b) PGSA30, (c) PGSA7-co-PEGDA=1:1, (d) PGSA15-co-PEGDA=1:1, (e) PGSA30-co-PEGDA=1:1, (f) PEGDA, (g) PGSA7-co-PCLDA=2:1, (h) PGSA15-co-PCLDA=2:1, (i) PGSA30-co-PCLDA=2:1 and (j) PCLDA.

Table S1 Thermal properties of DLP-AM printed PGSA, PCLDA, PEGDA, and copolymer films.

**Polymer**	**Ratio**	**Tg (°C)**	**Tm(°C)**
PGSA15	100%	N/A	15
PGSA30	100%	-17	N/A
PGSA7-co-PEGDA	1:1	-45	N/A
PGSA15-co-PEGDA	1:1	-38	N/A
PGSA30-co-PEGDA	1:1	-35	N/A
PEGDA	100%	-45	N/A
PGSA7-co-PCLDA	2:1	N/A	-37
PGSA15-co-PCLDA	2:1	-45	3
PGSA30-co-PCLDA	2:1	N/A	N/A
PCLDA	100%	N/A	30

1. Nijst, C.L.E.; Bruggeman, J.P.; Karp, J.M.; Ferreira, L.; Zumbuehl, A.; Bettinger, C.J.; Langer, R. Synthesis and Characterization of Photocurable Elastomers from Poly(glycerol-co-sebacate). *Biomacromolecules* **2007**, *8*, 3067-3073, doi:10.1021/bm070423u.
